# The Role of Medicine in Protecting the Vulnerable: between Sexual Violence and Conscientious Objection

**DOI:** 10.1055/s-0040-1721684

**Published:** 2020-11-30

**Authors:** Marcos Felipe Silva de Sá

**Affiliations:** 1 Faculdade de Medicina de Ribeirão Preto, Universidade de São Paulo, Ribeirão Preto, SP, Brazil

There are situations that cannot and should not be overlooked. Some situations must always be remembered so mistakes are not repeated and we seek to be better as people, professionals and citizens.

In August of this year, the case of a ten-year-old child in the southeastern region of Brazil that became pregnant with her uncle who had abused her since she was six was widely reported in the media. A child who lived in the company of her grandparents, and could say nothing about the rapes she suffered, given the risk of losing those who raised her. If she said anything, he [the uncle] would kill her grandfather. Four years of violence and silence. Four years of stolen childhood, until the symptom of abdominal pain caused her grandmother to take her to the hospital, where the pregnancy was revealed. What to do?

In Brazil, abortion is allowed by the legal system when there is no other way to save the pregnant woman's life; when the pregnancy results from rape; and in case of an anencephalic fetus pregnancy. These three cases do not require a court order, although an order to terminate the pregnancy had been issued by the Judge of the local Child and Youth Court after a request from the Public Ministry. However, the hospital, an accredited center for providing abortion care provided for by law in the region where the child lived, did not comply with the sentence under the allegation of “technical issues.”

This child and her grandmother were quickly taken to another referral center, this time in the northeast region, and finally, amid a great turmoil caused by pro-life and pro-choice movements, the abortion was performed by the hands of a competent and dedicated professional.

As a doctor, I do not have the knowledge to analyze legal issues, although it was clear that the Judiciary was present in this case and acted quickly to guarantee the dignity of life, paying attention to a basic principle of the Statute of the Child and Adolescent, which is their best interest, and protecting the right to health as well as the physical and psychological wellbeing.

While voices argued that pregnancy should be continued as if by a child's “moral obligation,” even though she had been abused and raped, others took the opposite position by defending her life in a broader sense: the biological and biographical life of a weeping girl in fear and pain while clung to a teddy bear. Fear and pain that would never be understood by police, judges, prosecutors, doctors, family members and an entire angry population.

Note the teachings of legal experts on this issue: “Childhood pregnancy, by itself, would already constitute a violation of a child's right. Would not the imposition of continuing this pregnancy be a further violation of her rights [...]?” We are talking about the pregnancy of “a ten-year-old girl, raped by someone from whom is expected care and protection.” 1

## What about Medicine?

The reasons raised by the referral center in the southeastern region for not having an abortion were that they followed the Ministry of Health guidelines that allow humanized abortion up to twenty-two weeks of pregnancy and the fetus' weight of up to five hundred grams. The law does not establish these limits. In this case, the fetus exceeded the estimated five hundred grams by thirty-seven grams. Because of this difference, the first referral center failed to comply with the court order. After all, is medicine an exact science? To what extent can a single argument like this be useful for medical decision making? Once abortion is permitted in specific cases, as provided by law, it is necessary to investigate if care centers accredited for this purpose are fulfilling their role.

In an article published in this issue of RBGO entitled “Conscientious objection to legal abortion in Minas Gerais State” 2 the authors clearly revealed the imperfections of the program created by the National Health System (Brazilian SUS) to assist patients in carrying out abortions provided for by law and involving as complex situations as the case above. In the referred work, data were collected from institutions accredited by the Health Department of the state of Minas Gerais (SES/MG) to assist sexual violence victims in the state (87 institutions). According to the results presented, although the services were accredited for this purpose, 11% of them do not have doctors to provide legal abortion and 31% do not provide specific training for this type of care. About 83% of patients willing to have a legal abortion who sought these services did not have their request responded. Of the reasons alleged for not having an abortion, the main one was the religious conscientious objection on the part of doctors working at the institution (57%).

The question of termination of pregnancy provided for by law is an unresolved situation in Brazil. Where these pregnant women should be referred and who should serve them are unanswered questions until today, as some hospitals refuse to perform these referrals and many professionals claim conscientious objection to avoid the commitment to carry out the interruption of pregnancy. How should doctors who will exercise conscientious objection in services aimed precisely at the care of women victims of sexual violence who seek abortion be accredited? When being a SUS professional and not working in a private clinic, is such an objection possible? Would not this hiring model enable the failure to comply with a legal determination in a biased way?

The conscientious objection foreseen in the Code of Medical Ethics is a right of doctors who refuse to perform procedures or services that are inconsistent with their religious, ethical, social, moral, and other convictions. Thus, pregnant women due to rape often do not receive adequate care and use clandestine clinics to terminate the pregnancy, thereby exposing themselves to potentially fatal risks, or continue with pregnancy, although undesired.

In this sense, the Ministry of Health has published the Technical Norm for the Prevention and Treatment of Diseases Resulting from Violence against Women and Adolescents, aiming to contribute to the planning and execution of actions that improve the quality of health care for the population suffering this type of offense. The Technical Norm issued by the Ministry of Health in 2005 states that “ *With respect to abortion, the Brazilian Government is a signatory to United Nations Conference documents that consider it a serious public health problem (Program of Action of the International Conference on Population and Development held in Cairo in 1994) and recommend that countries revise the laws penalizing the practice of unsafe abortion, that is, which poses risks to the life and health of women (Action Plan of the World Conference on Women held in Beijing in 1995). In this sense, it is necessary to guarantee the quality and expansion of referral services for performing the abortion provided for by law and to ensure that women who arrive at health services in the process of abortion are treated in a humanized manner, with care appropriate technology, thus avoiding the risk of illness and death”* . 3

To achieve these objectives, referral services were accredited by the SUS to provide this assistance to victimized patients, supported by the state, represented here by the institutions providing care. However, the issue of conscientious objection does not seem to have been properly considered by the SUS when accrediting a service with the premise to guarantee the performance of abortion provided for by law with quality and safety hence, without objector medical professionals in the team. In the aforementioned work, the authors reported that conscientious objection was the main cause of refusing assistance to patients in the accredited services evaluated. However, even if they were objectors, the patient embracement and clarification would be the professional's duty, as well as referral to services where the procedure would be effectively performed or else guarantee their care by another professional of the institution. Thus, the need to alert the objector professional who performs the care of these patients, as well as in all other medical situations when they should abstract from the motivating convictions of their conscientious objection and guide their conduct and attitudes for the benefit of patients, but never to their detriment or for shady purposes, as established in the good principles of Hippocratic medicine.

The doctor-patient relationship is no longer vertical and has been built through dialogue. “Of course, doctors are also a subject of this legal relationship, but their role is to collaborate with the main subject, and not to treat him/her as an object of rights. We cannot forget we live in a pluralistic society, with different cultural currents, and the critical judgment to human values deserves to be observed.” 4

The form of accreditation of referral centers of this nature should be revised by the SUS. These aspects must be taken into account, whether in the selection of personnel or in the choice of institutions hosting the services by prioritizing those synergistic with the program purposes under penalty of setting up true obstetric violence by default.

The report on the Referral Centers of Minas Gerais published in this issue of RBGO is only the “tip of the iceberg” of a wider and much more complex problem. The accreditation of referral services without due care with the preparation of accredited institutions, using political criteria for the allocation of scarce resources of the SUS will not solve the problem nor will improve the system. Brazilian women will remain unassisted most of the time. Recently proposed measures contained in Ordinance of the Ministry of Health number 2.561 of September 23, 2020, 5 such as how to communicate the fact to the responsible police authority; preserve possible material evidence of the crime of rape to be immediately handed over to the police authority or official experts; and include the anesthesiologist in the multidisciplinary health team, are just distracting measures to change the focus of the problem and hinder the performance of health professionals truly involved with these issues. The appreciation of already accredited institutions that adequately fulfill their mission in a safe, professional and, above all, humanized way would be a better measure.

In demanding times, when right and left extremism are gaining ground, there must be caution so we, medical professionals, do not make decisions based solely on our pre-understandings and moral conceptions. The Hippocratic oath taken on the graduation of doctors must always prevail: “I will not allow that considerations of religion, nationality, race, political party, or social position come between my duty and my Patient.” In the case of women and adolescents victims of sexual violence, the promotion of technical scientific knowledge combined with the awareness of health professionals and the accreditation of institutions truly engaged in this cause are the best options for the SUS toward raising the quality of care and providing a definitive solution to this admittedly serious public health problem in Brazil.
